# Response surface methodology-based optimization of *Inonotus hispidus*’ liquid fermentation medium and evaluation of its exopolysaccharide activities

**DOI:** 10.3389/fmicb.2024.1456461

**Published:** 2024-09-04

**Authors:** Yuhan Gao, Xiaomin Li, Hui Xu, Huijuan Sun, Junli Zhang, Xiaoping Wu, Junsheng Fu

**Affiliations:** ^1^College of Life Sciences, Fujian Agriculture and Forestry University, Fuzhou, China; ^2^Chemical and Biomolecular Engineering Department, College of Design and Engineering, National University of Singapore, Singapore, Singapore; ^3^Mycological Research Center, Fujian Agriculture and Forestry University, Fuzhou, China; ^4^Tibet Academy of Agricultural and Animal Husbandry Sciences, Lhasa, China

**Keywords:** *Inonotus hispidus*, exopolysaccharides (EPS), response surface optimization, antioxidation, anticancer

## Abstract

**Introduction:**

*Inonotus hispidus*, commonly referred to as the Sanghuang mushroom, is a species that is consumed as a tea. To date, this is the only species of the same fungus that has been successfully cultivated.

**Methods:**

A single-factor test was conducted using *Inonotus hispidus* MS-5 and MS-9 as test materials. The response surface methodology was adopted to design and optimise the liquid fermentation medium for them.

**Results:**

As indicated in the results, the optimum fermentation conditions for MS-5 include 24.09 g/L glucose, 7.88 g/L yeast extract, 0.99 g/L dandelion powder, 1.5 g MgSO_4_, 2 g KH_2_PO_4_, 0.01 g vitamin B_1_, and 1 L deionized water; the optimum fermentation conditions for MS-9 include 24.64 g/L glucose, 7.77 g/L yeast extract, 0.98 g/L dandelion powder, 1.5 g MgSO_4_, 2 g KH_2_PO_4_, 0.01 g vitamin B_1_, and 1 L deionized water. Under such conditions, the mycelial biomass (dry weight) values were able to reach 16.02 g/L and 14.91 g/L for MS-5 and MS-9, respectively, which were 1.6 and 1.54 times those measured before optimization.

**Discussion:**

As revealed in the antioxidant and anticancer experiment, *Inonotus hispidus* exopolysaccharides has corresponding functional effects at the cellular level. This research optimised the liquid culture formulation of *Inonotus hispidus* and demonstrated that the function of it as a traditional Sanghuang herbal tea is well-documented.

## 1 Introduction

*Inonotus hispidus* falls under genus *Inonotus*, family Hymenochaetaceae, order Hymenochaetales, class Agaricomycetes, and phylum Basidiomycota (Dai and Yang, 2008; Markakis et al., 2017). *Inonotus hispidus* has been regarded as a valuable edible fungus since ancient times. In 1996, the functional activity of the *Inonotus hispidus* fruiting body was reported for the first time: it was revealed that hispolon extracted from the fruiting body demonstrated strong antiviral and immune-modulating functions (Ali et al., 1996). In recent years, increasing application value of *Inonotus hispidus* extract has been uncovered; for instance, Chen et al. (2018) extracted polyphenols and flavonoids from the *Inonotus hispidus* fruiting body and found that it exhibited very high antioxidant and bacteriostatic activities. The active substance, (4S, 5S)-4-hydroxy-3,5-cyclohexanone diethyl acetal-2-ketene (HDE) (Yang et al., 2019), in the *Inonotus hispidus* fruiting body produces an apparent inhibiting effect on cancer cells HepG2; there are also certain inhibiting effects from Inonotusin A (Zan et al., 2011) on human breast cancer cells MCF-7, and from Inoscavin C (Zan et al., 2015) on ovarian adenocarcinoma cells skov3 and hepatoma carcinoma cells HepG2. Therefore, *Inonotus hispidus* is of significant value for medicinal applications as an edible fungus.

Polysaccharides are among the main active substances in *Inonotus hispidus*. Liu et al. (2019a) extracted polysaccharide components from the *Inonotus hispidus* fruiting body and mycelium, which demonstrated a protective effect on mice with acute alcoholic liver injury. Zhang and Bao (2014) found that the crude polysaccharide content in the *Inonotus hispidus* fruiting body was 4.1% and showed high antineoplastic activity in H22 tumor-bearing mice. The polysaccharide content in *Inonotus hispidus* also exhibits high antioxidant activity (Liu et al., 2019b). Currently, the polysaccharide content present in *Inonotus hispidus* is mainly extracted from the fruiting body or mycelium; however, the wild fruiting body of *Inonotus hispidus* features scarce resources, difficult cultivation, a long cultivation cycle, and high labor costs, making it difficult to quickly obtain components from the fungus, thus restricting further development and utilization of its polysaccharide products. Many studies have confirmed that the production of exopolysaccharides from macro fungi, utilizing fermentation technology, is a potential pathway toward obtaining active polysaccharides by replacing the fruiting body (Gao et al., 2019; Jia et al., 2017); however, the activity and output of exopolysaccharides are generally affected by fermentation conditions, so it is particularly important to optimize fermentation technology to produce polysaccharides from *Inonotus hispidus*.

With regard to the optimization of culture media, the selection of multiple carbon and nitrogen sources allows for the study of the utilization of different nutrients by specific microorganisms (Hamad et al., 2014; Farag et al., 1983; Dörsam et al., 2017). In order to more effectively control the variables, this study employed a synthetic culture medium as the basis for the research. In regard to the formulation of the culture medium, the objective was to guarantee the reproducibility and operability of the experiment. To this end, carbon and nitrogen sources that are commonly utilized in microbiological research were selected. The selected carbon sources are representative of those commonly utilized in culture media, encompassing monosaccharides, disaccharides, and alcohols. To more comprehensively encompass carbohydrates with diverse structures, the selection encompasses both pentose and hexose. Furthermore, in regard to nitrogen sources, factors such as commonality and ease of acquisition were also taken into account. Organic and inorganic nitrogen sources that are readily available and commonly utilized were selected for inclusion. The Response Surface Methodology (RSM) method is employed to facilitate the efficient experimentation and verification of culture medium formulations under complex conditions with multiple factors and levels, thereby markedly enhancing efficiency.

*Inonotus hispidus*, a Sanghuang mushroom species, can be cultivated on basswood; thus, there is a paucity of studies on the subject of liquid fermentation. Currently, there is a paucity of literature on the subject of polysaccharide production by liquid fermentation of *Inonotus hispidus*. The objective of this study was to enhance the efficiency and simplicity of the production steps of polysaccharides in *Inonotus hispidus* through the implementation of liquid fermentation. To achieve this, a combination of single-factor experiments and response surface methodology was employed to optimize the liquid fermentation medium of *Inonotus hispidus*. The objective is to obtain a substantial quantity of mycelium and extracellular polysaccharides from the fermentation broth; it also explores the antioxidant activity and antitumor activity of exopolysaccharides, thus providing a reference for the efficient utilization of those from *Inonotus hispidus* for the development of antioxidant and anticancer products.

## 2 Materials and methods

### 2.1 Preparation of test strains and culture media

Test strains: the strains of *Inonotus hispidus* (strain numbers MS-5 and MS-9) used in this test were both from the Mycological Research Center of Fujian Agriculture and Forestry University.

Solid culture medium: 200 g potatoes (peeled), 20 g glucose, 2% agar, 5 g peptone, 1.5 g MgSO_4_, 2 g KH_2_PO_4_, and 0.01 g vitamin B_1_ (added after sterilization of the culture medium); deionized water was used to calibrate to 1 L, and the medium was bottled and sterilized for later use. In the following text, the medium is referred to as “potato dextrose agar (PDA).”

Basic fermentation medium: 200 g potatoes (peeled), 20 g glucose, 5 g peptone, 1.5 g MgSO_4_, 2 g KH_2_PO_4_, and 0.01 g vitamin B_1_ (added after sterilization of the culture medium); deionized water was used to calibrate to 1 L, and the medium was bottled and sterilized for later use. In the following text, the medium is referred to as “potato dextrose broth (PDB).”

### 2.2 Strain activation and shake flask culture

Strain activation: *Inonotus hispidus* strains were inoculated onto the plate, cultured at a constant temperature of 25°C, and then taken out for future use once hyphae grew throughout the plate.

Shake flask culture: Five 7 mm diameter fungal clusters were incubated individually in flasks containing 100 mL basic fermentation medium. Culture conditions of the shake flask: 25°C, 160 rpm, not exposed to light, and cultured for 15 days for future use.

### 2.3 Single-factor test

Two variables, i.e., a carbon source and a nitrogen source, were studied as the basis of a basal fermentation medium. In the single-factor carbon source test, the carbon source was taken as the variable, while other factors remained unchanged, and the single factors recorded were maltose, saccharose, glucose, fructose, xylose, mannitol, mannose, lactose, and galactose; the added amount for each of them was 2%, and the absence of an added carbon source was taken as the control. In the single-factor nitrogen source test, the nitrogen source was taken as the variable, while other factors were unchanged, and single factors recorded were yeast extract, peptone, ammonium tartrate, ammonium sulfate, ammonium nitrate, urea, and beef extract; the added amount for each of them was 0.5%, and the absence of an added nitrogen source was taken as the control. Single-factor culture conditions were the same as those specified above, in Section 2.2. The impacts of different individual factors on the biomass of *Inonotus hispidus* were studied.

### 2.4 Screening test on the optimum added quantities of carbon and nitrogen sources, and on exogenous substances

In order to identify the optimum quantities for the carbon and nitrogen sources, a single-factor test was carried out. The researched quantities of an added carbon source were set as 10, 15, 20, 25, and 30 g/L, while those of an added nitrogen source were set as 2.5, 5, 7.5, 10, and 15 g/L; subsequently, the screening tests to determine the optimum added quantities were carried out. Three repetitions were set for each group; after the culture was completed, the determination of mycelial biomass was conducted, with the culture conditions and determination method identical to those specified in Section 2.2, above.

As revealed in the previous test, dandelion powder can significantly promote the growth of *Inonotus hispidus*. Therefore, dandelion powder was added, as an exogenous substance, to the basal fermentation medium, and the gradient was set to 0, 0.5, 0.75, 1, 1.25, and 1.5 g/L; thereafter, the screening tests on the optimum added quantities were carried out. For each group, the process was repeated three times. Once the culture was complete, we determined the mycelial biomass using the same conditions and methodology outlined in Section 2.2.

### 2.5 Response surface optimization test for box-Behnken design

According to the results of the single-factor tests and the screening tests on the optimum added quantities, response surface analysis was conducted, with the aim of exploring the relationships between the independent and dependent variables. To this end, the Box-Behnken central composite design principle was employed in the context of response surface methodology (Li et al., 2021). Three potential influencing factors were considered, including the carbon and nitrogen sources, as well as the potential benefit of incorporating dandelion powder, for which a 3-factor 3-level test was conducted for low, medium, and high levels—set as −1, 0, and 1—of each independent variable; with the mycelial biomass as the response value, optimization design and analysis were carried out.

### 2.6 Verification test

With the mycelial biomass as the response value, optimization was achieved using response surface software to predict the optimum fermentation conditions, after which a verification test was conducted according to the predicted optimum combination.

### 2.7 Determination of mycelial biomass

After culture in the liquid spawn shake flask was completed, fermentation broth underwent suction filtration using non-woven fabrics; mycelia were washed with sterile water several times, then dried at 65°C to constant weight, before being weighed with an electronic scale.

### 2.8 Preparation of exopolysaccharides

Preparation of exopolysaccharides: After fermentation, the fermentation broth underwent vacuum filtration and was centrifuged at 10,000 rpm for 3 min, after which it was concentrated, via rotary evaporation, to 1/5 of the original volume; with the addition of ethyl alcohol at four times the volume of the concentrated solution, it then underwent alcohol precipitation at 4°C overnight. Centrifugal collection and precipitation followed, and water was added for redissolution; after deproteinization via the Sevag method (Bai et al., 2020), diethyl ether and methyl alcohol, among others, were added to remove impurities. Finally, flowing water dialysis was performed for 48 h; after freeze drying, a polysaccharide sample was collected and kept at −20°C for future use.

### 2.9 Chemical antioxidant activity of exopolysaccharides

Determination of ABTS (2,2′-Azinobis-(3-ethylbenzthiazoline-6-sulphonate)) free radical scavenging ability: An ABTS scavenging method (Hu et al., 2017) was adopted to determine the ABTS free radical scavenging ability, and the same concentration of vitamin C was utilized as a positive control group (hereinafter referred to as VC) for the purpose of comparing the antioxidant capacity. ABTS free radical scavenging rate (%) = [1 − (*A_x_* − *A*_*x*0_)/*A*_0_] × 100, where *A_x_* is the sample’s light absorption value, *A*_*x*0_ is the light absorption value based on the replacement of ABTS with distilled water, and *A*_0_ is the light absorption value based on replacement of the sample with distilled water.

Determination of DPPH (1,1-Diphenyl-2-picrylhydrazyl radical 2,2-Diphenyl-1-(2,4,6-trinitrophenyl) hydrazyl) free radical scavenging ability: A DPPH scavenging method (Xie et al., 2019) was adopted to determine the DPPH free radical scavenging ability, and V_C_ was taken as the positive control group. DPPH free radical scavenging rate (%) = [1 − (*A_x_* − *A*_*x*0_)/*A*_0_] × 100, where *A_x_* is the sample’s light absorption value, *A*_*x*0_ is the light absorption value based on the replacement of DPPH with a 95% ethanol solution, and *A*_0_ is the light absorption value based on the replacement of the sample with distilled water.

Determination of total antioxidant capacity (ferric ion reducing antioxidant power, FRAP): Reference was made to the method developed by Li et al. (2018), which was slightly modified. A 30 μL sample solution, with different concentrations, was taken and added to a 180 μL FRAP working solution (0.3 mol/L pH 3.6 sodium acetate buffer solution, 10 mmol/L TPTZ solution, and 20 mmol/L FeCl_3_ solution were mixed at a ratio of 10:1:1 (available for use upon preparation)); five repetitions were set for each group, and incubation lasted for 20 min at 25°C, after which the light absorption value was measured at 593 nm using a microplate reader. With the sample replaced with FeSO_4_ standard solution, subject to (0.1–2.0) mmol/L concentration, a standard curve was drawn, with the concentration and absorbency as the *x*- and *y*-coordinates, respectively, thus arriving at the following regression equation: *y* = 0.4839*x* − 0.2152; correlation coefficient *R*^2^ = 0.9991. According to the *A* value after reactions (*A* value = *A_x_* − *A*_0_ − *A*_*x*0_, *A_x_* is the sample’s light absorption value, *A*_*x*0_ is the light absorption value based on the replacement of DPPH with a 95% ethanol solution, and *A*_0_ is the light absorption value based on the replacement of the sample with distilled water), the corresponding FeSO_4_ concentration (mmol/L) was obtained through the standard curve, and it was defined as the FRAP value; as the value increases, the antioxidant activity also increases.

### 2.10 Determination of exopolysaccharide anticancer ability

MTT cell proliferation assay: (1) Cell spreading on plates: cell growth was microscopically observed; when it reached the logarithmic phase, cells were spread on plates. Action was taken to suck out cell culture fluid, then cells were washed twice with PBS buffer solution and added to a 600 μL pancreatin solution, after which the solution was digested within an incubator. When cell floating was observed, 5 mL cell culture fluid was added to terminate digestion, and the sample was transferred to a centrifugal tube to be centrifuged at 1,000 rpm for 3 min. After the supernatant was removed, cell culture fluid was added and cells were gently blown, thus uniformly dispersing the cells. Then, 200 μL cancer cells were inoculated onto 96-well plates, with an outer ring of PBS buffer solution added to mitigate volatilization of the cell culture fluid. The 96-well plates onto which cells had been spread were placed into a 37°C, 5% carbon dioxide incubator for culture; 24 h later, they were observed to check whether cell adherence had occurred (Liu et al., 2020). (2) Cell-based drug delivery: Experimental, blank, and control groups were established; once observation showed cell adherence and growth, care was exercised to suck out cell culture fluid. In the experimental group, different exopolysaccharide samples were made into solutions with cell culture fluid, at concentrations of 0.025, 0.05, 0.25, 0.5, 1, 2, and 5 mg/mL, with 200 μL added into each well and five repetitions performed. In the blank group, no cells were spread, and cell culture fluid was added; cell culture fluid and cells were added to the control group, and 96-well plates to which drugs had been delivered were cultured in a 37°C, 5% carbon dioxide incubator for 24 h. (3) MTT colorimetric method: Before experimentation, PBS buffer solution was added into a 5 mg/mL MTT solution; it was sieved using a filter head, not exposed to light, and kept in a refrigerator at 4°C for future use. After growth for 24 h on 96-well plates to which drugs had been delivered, the contents of each well were aspirated, and then an aliquot of 10 μL MTT solution and 90 μL cell culture fluid was added; after culture within an incubator for 4 h, the culture solution was carefully removed from each well, and 150 μL DMSO was added. It was vibrated for 15 min to sufficiently dissolve formazan, and a microplate reader was used to detect each well’s light absorption value (OD value) at a 490 nm wavelength.

### 2.11 Data analysis

SPSS 26.0 and GraphPad Prism5.0 software were adopted to conduct one-way analysis of variance (ANOVA); LSD was used to test the significance of analysis, for which *p* < 0.05 denotes a significant difference, while *p* < 0.01 indicates a highly significant difference.

## 3 Results

### 3.1 The impacts of single factors on the mycelial biomass of *Inonotus hispidus*

#### 3.1.1 The impacts of different carbon sources and their dosages on the mycelial biomass of *Inonotus hispidus*

**Figure 1:**
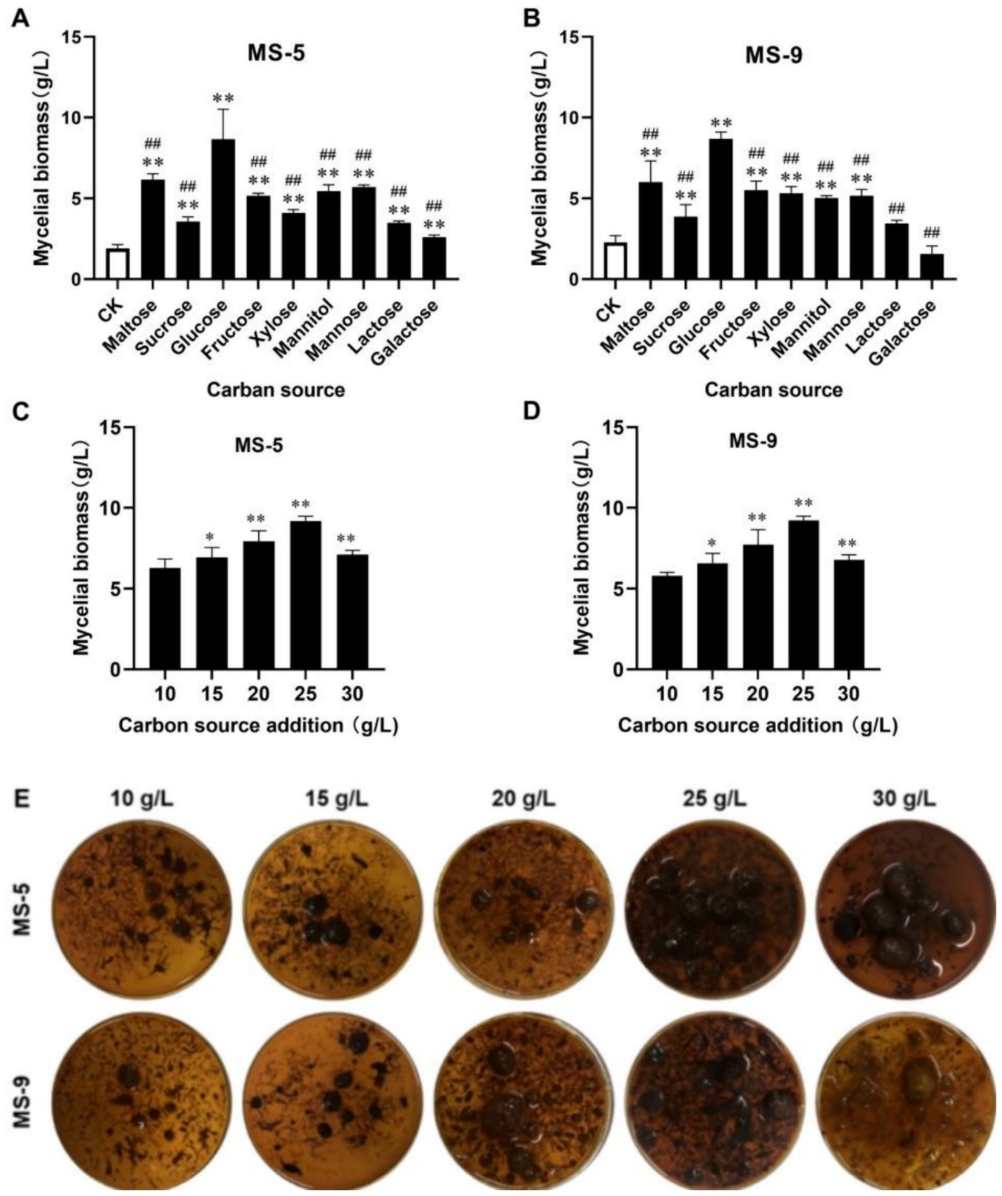
The impacts of different carbon sources and their dosages on the mycelial biomass of *Inonotus hispidus*. Each experiment was conducted five times. **(A)** The influences of diverse carbon sources on the mycelial biomass of MS-5; **(B)** the impacts of different carbon sources on MS-9’s mycelial biomass; **(C)** the impacts of different glucose dosages on MS-5’s mycelial biomass; **(D)** the impacts of different glucose dosages on MS-9’s mycelial biomass; **(E)** fermentation broth for *Inonotus hispidus* at different glucose dosages. In the carbon source screening test, compared with the CK group, ^*^p < 0.05 and ^**^p < 0.01; compared with the optimum carbon source, ^#^*p* < 0.05 and ^##^*p* < 0.01. In the carbon source dosage screening test, compared with the previous group, ^*^p < 0.05 and ^**^p < 0.01.

As shown in Figures 1A,B, when the carbon source is glucose, the mycelial biomass of *Inonotus hispidus* can be significantly increased to 4.82 times that of the control group; meanwhile, when the carbon sources are saccharose and galactose, the increases are 2.33 times and 1.12 times that of the control group, respectively; the remaining carbon sources can, to some extent, promote mycelial growth, but the effect from glucose is the most evident, and with this carbon source, the MS-5 and MS-9 mycelial biomass levels reach 8.67 g/L and 8.68 g/L, respectively, more than two times those of the blank group (CK). Therefore, glucose is the optimum carbon source for liquid fermentation of *Inonotus hispidus*.

As indicated in Figures 1C–E, with an increasing quantity of added glucose, the mycelial biomass of *Inonotus hispidus* rises; when the quantity of added glucose reaches 25 g/L, the mycelial biomasses of *Inonotus hispidus* MS-5 and MS-9 reach their maximum values, of 9.2 g/L and 9.23 g/L, respectively; when the quantity of added glucose continues to increase, the mycelial biomass levels decline somewhat. Therefore, 25 g/L is the optimum quantity of added glucose for the liquid fermentation medium of *Inonotus hispidus*.

#### 3.1.2 The impacts of nitrogen sources on the mycelial biomass of *Inonotus hispidus*

**Figure 2:**
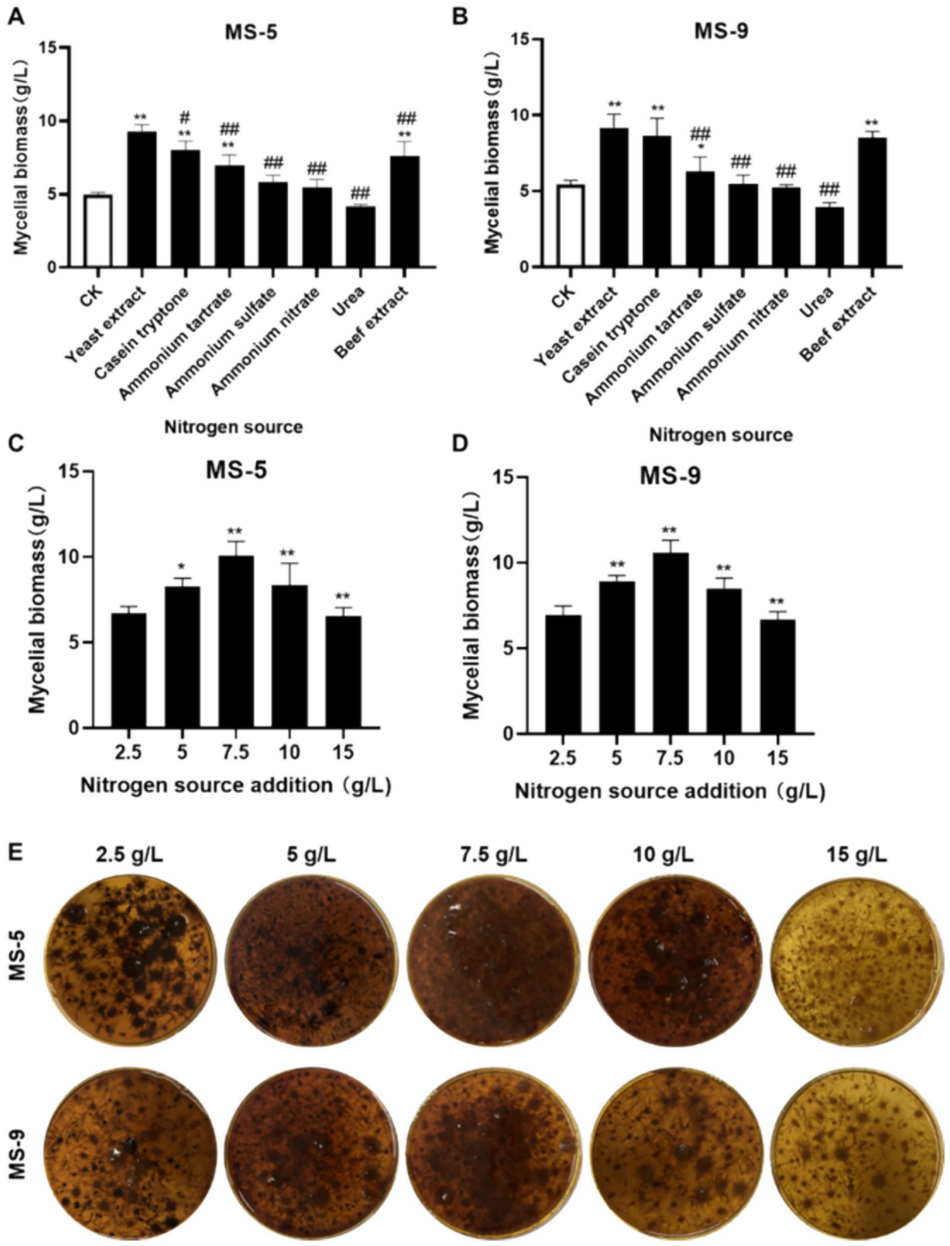
The impacts of different nitrogen sources and their dosages on the mycelial biomass of *Inonotus hispidus*. Each experiment was conducted five times. **(A)** The influences of diverse nitrogen sources on the mycelial biomass of MS-5; **(B)** the impacts of different nitrogen sources on MS-9’s mycelial biomass; **(C)** the impacts of different yeast extract dosages on MS-5’s mycelial biomass; **(D)** the impacts of different yeast extract dosages on MS-9’s mycelial biomass; **(E)** fermentation broth for *Inonotus hispidus* at different yeast extract dosages. In the nitrogen source screening test, compared with the CK group, ^*^p < 0.05 and ^**^p < 0.01; compared with the optimum nitrogen source, ^#^*p* < 0.05 and ^##^*p* < 0.01. In the nitrogen source dosage screening test, compared with the previous group, ^*^p < 0.05 and ^**^p < 0.01.

As shown in Figures 2A,B, it is very easy to conclude that the effects of yeast extract, peptone, and beef extract on promoting growth are highly significant; when yeast extract is used, the biomass levels of MS-5 and MS-9 are 9.27 g/L and 9.17 g/L, respectively, which are more than 1.29–1.86 times those of the control group (CK), while the roles of four nitrogen sources, i.e., ammonium tartrate, ammonium sulfate, ammonium nitrate, and urea, in promoting the growth of *Inonotus hispidus*’ mycelial biomass are not significant enough. Therefore, yeast extract is also the optimum nitrogen source for the liquid fermentation of *Inonotus hispidus*.

As indicated in Figures 2C–E, when the added quantity of yeast extract is 7.5 g/L, the mycelial biomass of *Inonotus hispidus* reaches its highest level, while the mycelial biomass levels of MS-5 and MS-9 reach 10.06 g/L and 10.56 g/L, respectively, which are 1.18 and 1.21 times the original ones, compared with an added nitrogen source of 5 g/L for ordinary PDB medium. Therefore, the optimal quantity of added yeast extract for the liquid fermentation of *Inonotus hispidus* is determined to be 7.5 g/L.

### 3.2 Screening for the optimum dosage of exogenous growth factors

**Figure 3:**
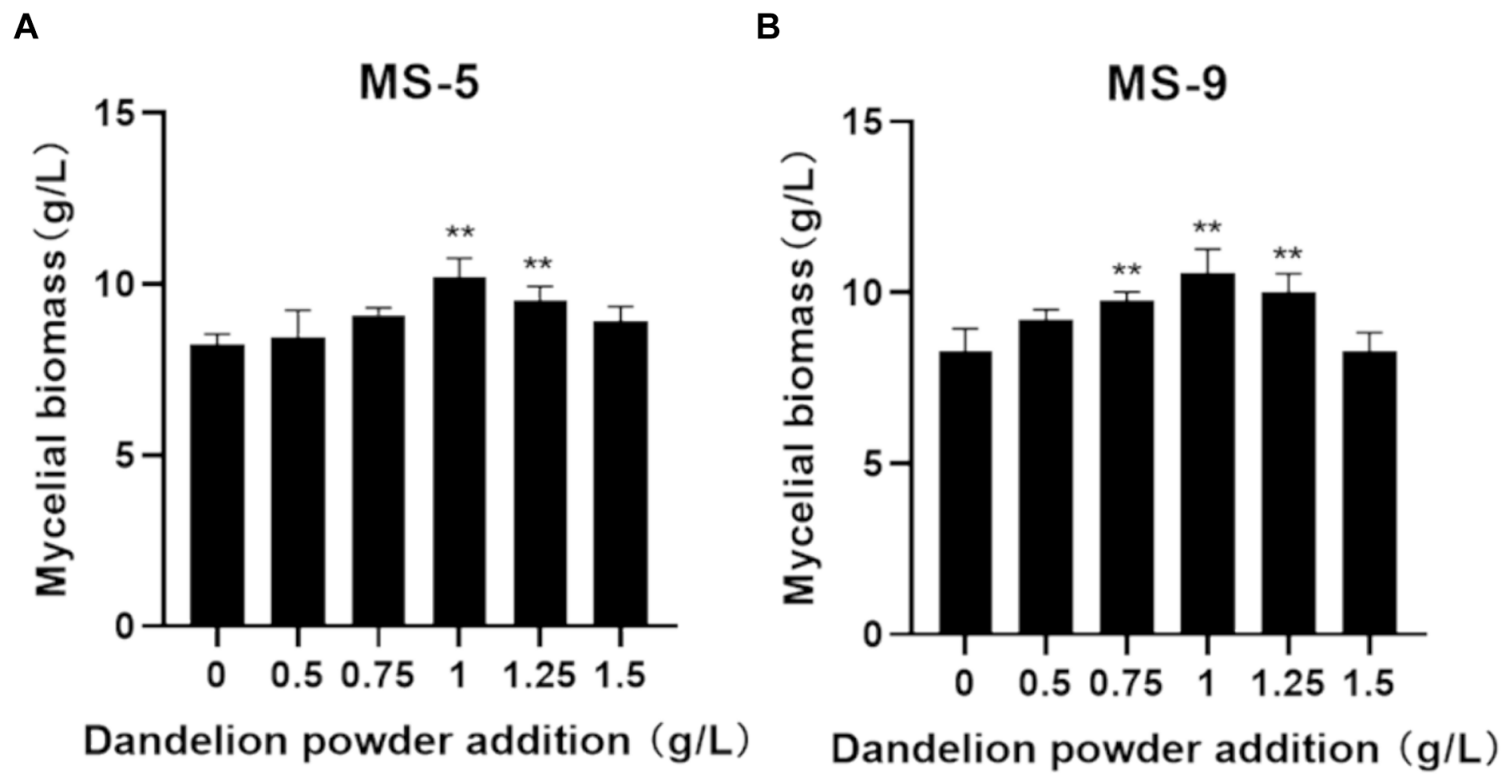
The impact of the quantity of added dandelion powder on the mycelial biomass of *Inonotus hispidus*. Compared with the blank group, ^*^p < 0.05 and ^**^p < 0.01. Each experiment was conducted five times. **(A)** is the mycelial biomass of the MS-5 strain of Inonotus hispidus after 15 days of culture; **(B)** is the mycelial biomass of the MS-9 strain of Inonotus hispidus after 15 days of culture.

According to the results involving the impact of adding dandelion powder to the liquid fermentation process for *Inonotus hispidus*, as shown in Figure 3, the role of the low-dosage (0.5 g/L) added quantity in promoting *Inonotus hispidus*’ growth is not significant; when the added quantity increases to 1 g/L, the mycelial biomass significantly increases compared with that of the blank group, and MS-5’s and MS-9’s mycelial biomass levels become 1.23 and 1.27 times those when dandelion powder is not added; as the added quantity continues to increase, the promoting effect declines somewhat. Therefore, the addition of 1 g/L dandelion powder for the liquid fermentation process of *Inonotus hispidus* can effectively promote mycelial growth.

### 3.3 The design and result of the *Inonotus hispidus* response surface experiment

#### 3.3.1 The design and result of the MS-5 response surface experiment

The experimental design and results are shown in Table 1. The fitted quadratic polynomial, obtained by taking mycelial biomass as the response value, is as follows: *Y* = 15.902 − 0.66625A +  0.69375B − 0.135C − 1.115AB − 0.4825AC − 0.4575BC − 2.2735A^2^ − 3.0135B^2^ − 2.831C^2^. The quadratic polynomial variance and significance of the response surface analysis are shown in Table 2; the result of *p* < 0.0001 suggests that the model is highly significant. In this case, A, B, AB, A^2^, B^2^, and C^2^ are model items; the lack-of-fit *F* value of 2.69 implies that the lack of fit is not significant with respect to pure error, and the lack-of-fit *p* = 0.1817 is not significant; therefore, the model can be used to predict and analyze the optimum conditions for the liquid fermentation of MS-5’s mycelial biomass.

**Table 1:** Response surface design and test results for MS-5.

No.	A: Carbon source (g/L)	B: Nitrogen source (g/L)	C: Dandelion powder (g/L)	Mycelial biomass (g/L)
1	25	10	0.75	11.36
2	25	5	1.25	9.67
3	30	10	1	9.43
4	25	7.5	1	15.83
5	25	7.5	1	16.16
6	20	7.5	0.75	10.63
7	25	10	1.25	9.77
8	30	7.5	1.25	10
9	20	10	1	13.56
10	20	7.5	1.25	11.73
11	25	7.5	1	15.46
12	25	5	0.75	9.43
13	30	7.5	0.75	10.83
14	25	7.5	1	16.43
15	20	5	1	9.57
16	30	5	1	9.9
17	25	7.5	1	15.63

**Table 2:** Variance analysis and significance tests for MS-5.

Source	Sum of squares	Df	Mean squares	*F*-value	*p*-value
Model	118.85	9	13.21	49.45	<0.0001
A	3.55	1	3.55	13.30	0.0082
B	3.85	1	3.85	14.42	0.0067
C	0.1458	1	0.1458	0.5459	0.4840
AB	4.97	1	4.97	18.62	0.0035
AC	0.9312	1	0.9312	3.49	0.1041
BC	0.8372	1	0.8372	3.13	0.1199
A2	21.76	1	21.76	81.49	<0.0001
B2	38.24	1	38.24	143.17	<0.0001
C2	33.75	1	33.75	126.35	<0.0001
Residual	1.87	7	0.2671		
Lack of fit	1.25	3	0.4166	2.69	0.1817
Pure error	0.6199	4	0.1550		
Cor Total	120.72	16			

*R2 = 0.9845; adjusted R2 = 0.9646; predicted R2 = 0.8264.*

**Figure 4:**
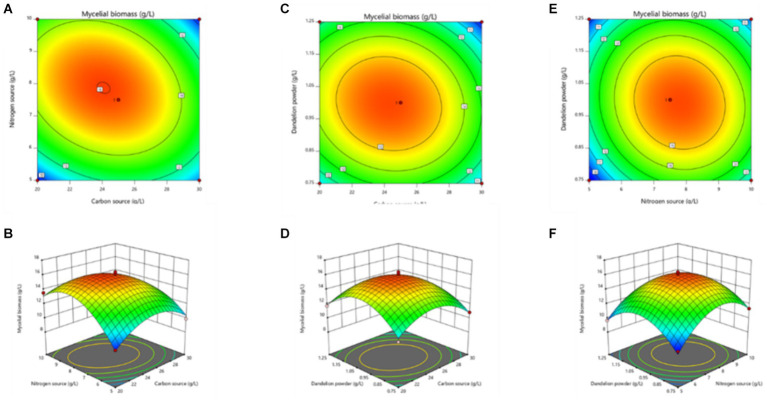
Response surface stereogram, involving Box-Behnken design optimization of mycelial biomass from MS-5 liquid fermentation. **(A)** represents the isoline value; **(B)** represents the interaction between the quantity of carbon source added and the quantity of nitrogen source added; **(C)** represents the corresponding isoline value; **(D)** represents the interaction between the quantity of carbon source added and the quantity of dandelion powder added; **(E)** represents the corresponding isoline value; **(F)** represents the interaction between the quantity of nitrogen source added and the quantity of dandelion powder added.

The curved response surface, generated through regression of the quadratic polynomial, as shown in Figure 4, reflects the impacts of three variables and their interactions on the mycelial biomass. The optimum liquid fermentation conditions, predicted with the response surface model, are as follows: added carbon source, 24.09 g/L; added nitrogen source, 7.88 g/L; added dandelion powder, 0.99 g/L. Under such conditions, MS-5’s mycelial biomass is expected to reach 16.02 g/L.

#### 3.3.2 The design and result of the MS-9 response surface experiment

The experimental design and results are shown in Table 3. The fitted quadratic polynomial obtained, by taking mycelial biomass as the response value, is as follows: *Y* = 14.864 − 0.28A + 0.5375B − 0.2825C − 0.3675AB − 0.0575AC − 0.3425BC − 2.2932A^2^ − 2.6582B^2^ − 2.58325C^2^. The quadratic polynomial variance and significance of the response surface analysis are shown in Table 4; the result of *p* < 0.0001 suggests that the model is highly significant. A, B, C, AB, A^2^, B^2^, and C^2^ are model items; the lack-of-fit *F* value 0.1199 implies that the lack of fit is not significant with respect to pure error, and the lack-of-fit *p* = 0.9437 is not significant; therefore, the model can be used to predict and analyze the optimum conditions for the liquid fermentation of MS-9’s mycelial biomass.

**Table 3:** Response surface design and test results for MS-9.

No.	A: Carbon source (g/L)	B: Nitrogen source (g/L)	C: Dandelion powder (g/L)	Mycelial biomass (g/L)
1	30	10	1	9.86
2	20	10	1	11.1
3	20	5	1	9.23
4	20	7.5	0.75	10.56
5	30	7.5	1.25	9.53
6	25	7.5	1	14.93
7	25	5	0.75	9.13
8	25	10	0.75	10.83
9	25	5	1.25	9.1
10	25	10	1.25	9.43
11	25	7.5	1	15.36
12	20	7.5	1.25	10.03
13	30	7.5	0.75	9.83
14	25	7.5	1	14.7
15	25	7.5	1	14.33
16	30	5	1	9.46
17	25	7.5	1	15

**Table 4:** Variance analysis and significance tests for MS-9.

Source	Sum of squares	Df	Mean squares	*F*-value	*p*-value
Model	93.95	9	10.44	114.33	<0.0001
A	0.6272	1	0.6272	6.87	0.0344
B	2.31	1	2.31	25.31	0.0015
C	0.6385	1	0.6385	6.99	0.0332
AB	0.5402	1	0.5402	5.92	0.0453
AC	0.0132	1	0.0132	0.1448	0.7148
BC	0.4692	1	0.4692	5.14	0.0577
A2	22.14	1	22.14	242.51	<0.0001
B2	29.75	1	29.75	325.84	<0.0001
C2	28.10	1	28.10	307.72	<0.0001
Residual	0.6392	7	0.0913		
Lack of fit	0.0582	3	0.0194	0.1337	0.9350
Pure error	0.5809	4	0.1452		
Cor Total	94.59	16			

*R2 = 0.9932; adjusted R2 = 0.9846; predicted R2 = 0.9806.*

**Figure 5:**
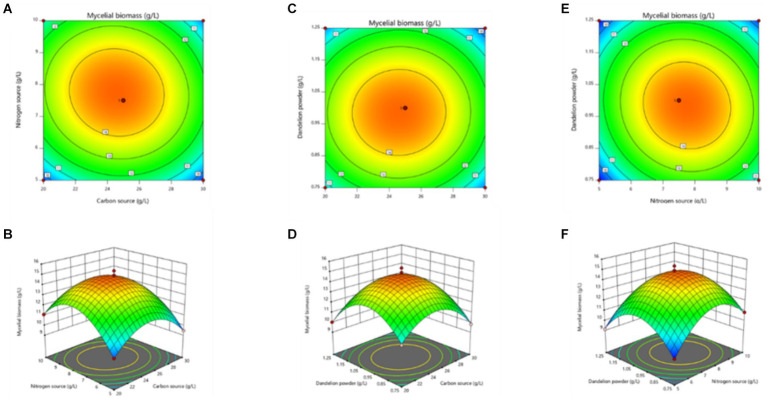
Response surface stereogram, involving Box-Behnken design’s optimization of mycelial biomass from MS-9 liquid fermentation. **(A)** represents the isoline value; **(B)** represents the interaction between the quantity of carbon source added and the quantity of nitrogen source added; **(C)** represents the corresponding isoline value; **(D)** represents the interaction between the quantity of carbon source added and the quantity of dandelion powder added; **(E)** represents the corresponding isoline value; **(F)** represents the interaction between the quantity of nitrogen source added and the quantity of dandelion powder added.

The curved response surface generated through regression of the quadratic polynomial, as shown in Figure 5, reflects the impacts of three variables and their interactions on the mycelial biomass. As revealed in the analysis, the optimum liquid fermentation conditions, predicted with the response surface model, are as follows: added carbon source, 24.64 g/L; added nitrogen source, 7.77 g/L; added dandelion powder, 0.98 g/L. Under such conditions, MS-9’s mycelial biomass is expected to reach 14.91 g/L.

### 3.4 The results involving verification of the optimum fermentation medium for *Inonotus hispidus*

**Figure 6:**
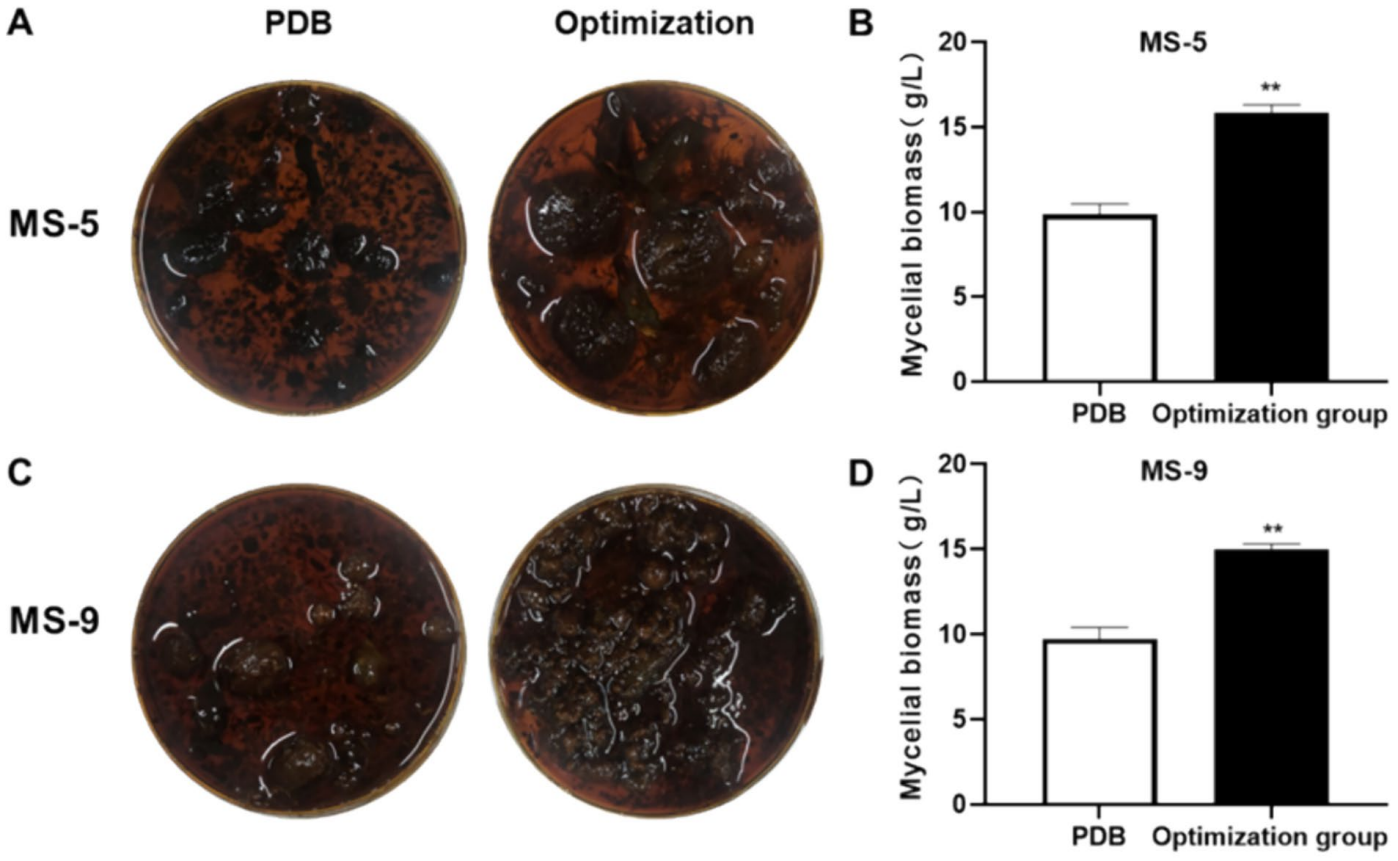
*Inonotus hispidus* liquid fermentation optimization formula verification experiment. Compared with the PDB group, ^*^p < 0.05 and ^**^p < 0.01. **(A)** is an overhead photo of the mycelium mass of the MS-5 strain after one cycle of cultivation in the culture medium before and after the improvement. **(B)** is a comparison of the corresponding weight (dry weight) of the MS-5 strain. **(C)** is an overhead photo of the mycelium mass of the MS-9 strain after one cycle of cultivation in the culture medium before and after the improvement. **(D)** is a comparison of the corresponding weight (dry weight) of the MS-9 strain.

The model’s accuracy was verified by predicting the liquid fermentation culture conditions for *Inonotus hispidus*; the mycelia were dried and weighed following the application of the fermentation culture. According to the experimental results shown in Figure 6, the MS-5 PDB group produced 9.86 ± 0.6 g/L mycelia, while, in the optimization group, mycelial weight was determined to be 15.82 ± 0.49 g/L, approximately 1.6 times that observed before the optimization process. The observed value exhibited a minimal discrepancy relative to the predicted value of 16.02 g/L; in the MS-9 PDB group, mycelia weighed 9.73 ± 0.68 g/L, while mycelia collected after optimization were 15.02 ± 0.31 g/L, 1.54 times that before optimization and slightly larger than the predicted value 14.91 g/L. Overall, the experimental prediction model is relatively rational.

### 3.5 Evaluation of the antioxidant activity of *Inonotus hispidus* exopolysaccharides

**Figure 7:**
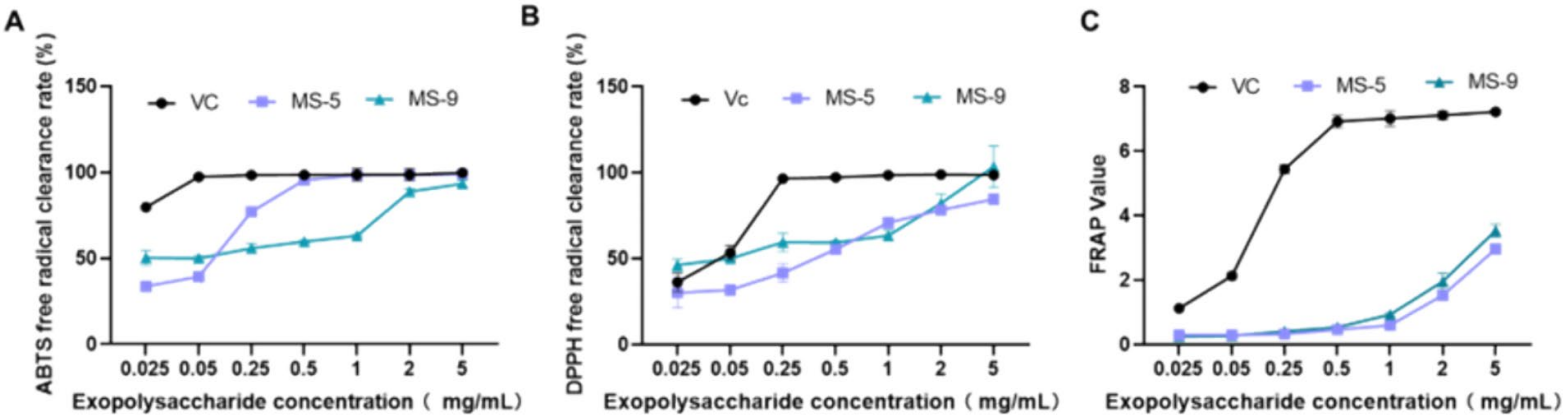
The antioxidant activities of *Inonotus hispidus* polysaccharides. **(A)** ABTS free radical scavenging capability; **(B)** DPPH free radical scavenging capability; **(C)** total antioxidant capability. Each experiment was conducted five times.

The antioxidant abilities of *Inonotus hispidus* MS-5 and MS-9 are shown in Figure 7 and Table 3. As shown in the experimental results, both MS-5 and MS-9 demonstrate antioxidant abilities; overall, MS-9’s free radical scavenging ability is higher than that of MS-5, and MS-9 exhibits higher antioxidant activity. For ABTS free radicals, MS-5 and MS-9 have similar antioxidant abilities, and MS-5’s EC_50_ is 0.058 mg/mL, while MS-9’s EC_50_ is 0.056 mg/mL, and MS-9’s scavenging ability is slightly higher than MS-5’s; for DPPH free radicals, MS-9’s antioxidant activity is higher than MS-5’s, which is consistent with the ABTS antioxidant result, but MS-9’s scavenging ability is much higher than MS-5’s; MS-9’s EC_50_ is only 0.067 mg/mL, while MS-5’s EC_50_ is 0.231 mg/mL, more than three times that of MS-9. In the FRAP experiment, MS-5 and MS-9 show similar antioxidant abilities; when the polysaccharide concentration is 5 mg/mL, its FRAP values are 2.96 ± 0.15 and 3.53 ± 0.19, with MS-9’s activity slightly higher than MS-5’s.

### 3.6 Evaluation of the anticancer cell activities of *Inonotus hispidus* polysaccharides

**Figure 8:**
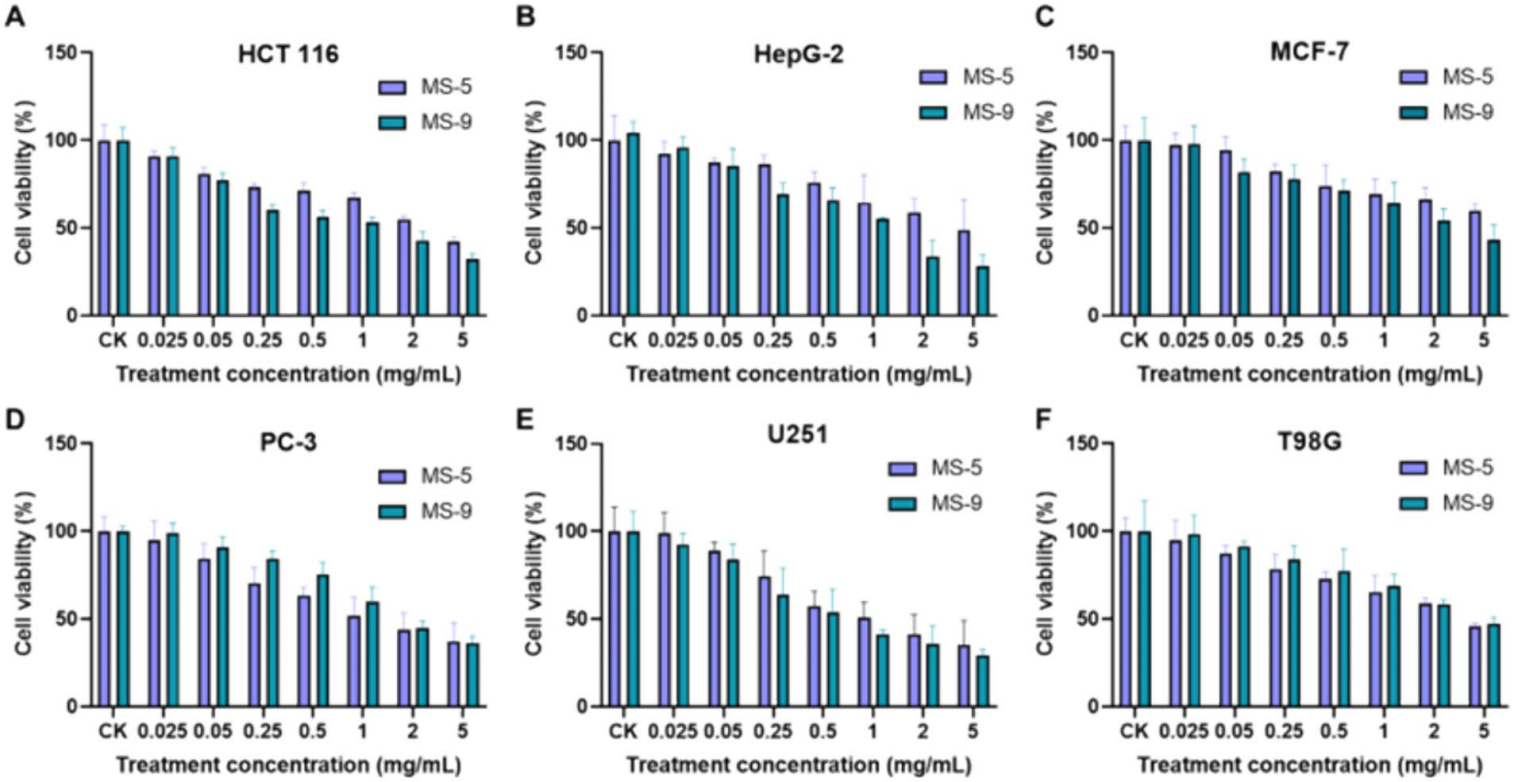
The effects of *Inonotus hispidus* exopolysaccharides on cancer cell growth. **(A)** The effect of extracellular polysaccharides of MS-5 and MS-9 on HCT 166. **(B)** The effect of extracellular polysaccharides of MS-5 and MS-9 on HepG-2. **(C)** The effect of extracellular polysaccharides of MS-5 and MS-9 on MCF-7. **(D)** The effect of extracellular polysaccharides of MS-5 and MS-9 on PC-3. **(E)** The effect of extracellular polysaccharides of MS-5 and MS-9 on U251. **(F)** The effect of extracellular polysaccharides of MS-5 and MS-9 on T98G.

The anticancer activities of *Inonotus hispidus* MS-5 and MS-9 exopolysaccharides in an *in vitro* setting for six cancer cell types is shown in Figure 8. According to the experimental results, *Inonotus hispidus* produces strong apoptosis-promoting effects on six cancer cell types, with the apoptosis-promoting effect on human neurogliocytoma cells U251 being the strongest among them. Different from glioma T98G, MS-9 *Inonotus hispidus* produces the most significant apoptosis-promoting effect; in the case of the highest concentration, the cell viability is only 29.38%, and its median inhibitory concentration is 0.738 mg/mL, while the apoptosis-promoting effect produced with MS-5 *Inonotus hispidus* is weaker; in the case of the highest concentration, 5 mg/mL, the cell viability is 35.15%. *Inonotus hispidus* has a weaker anticancer effect on human breast cancer cells MCF-7, with cancer cell viability between 43.49 and 44.97% and the IC_50_ values of MS-5 and MS-9 at 3.53 mg/mL and 3.235 mg/mL, respectively. When hepatoma carcinoma cells HepG-2 are treated with exopolysaccharides, MS-9 produces the best anticancer effect, with an IC_50_ value of only 1.169 mg/mL. When the polysaccharide concentration reaches 5 mg/mL, the cell viability is 28.08%, suggesting that MS-9 exopolysaccharides have strong apoptosis-promoting effects on hepatoma carcinoma cells HepG-2; while MS-5’s antitumor activity is moderate, its IC_50_ value is more than four times MS-9’s, reaching 4.687 mg/mL. MS-9 polysaccharides produce strong apoptosis-promoting effects on colon cancer cells HCT 116, and, compared with other strains, it has been found to exert significant inhibitory effects on *in vitro* cancer cell growth, with an IC_50_ value of 0.963 mg/mL; however, MS-5’s effect is weaker than MS-9’s, with an IC_50_ value of 2.129 mg/mL, more than two times MS-9’s. MS-5 has a better anticancer effect on human prostate cancer cells PC-3, and its apoptosis-promoting effect increases with concentration; in the case of the highest concentration in the experimental group, the cancer cell viability is 37.32%, and the IC_50_ value of MS-9 is 1.40 mg/mL, weaker than MS-5’s, with an IC_50_ value of 1.937 mg/mL. *Inonotus hispidus* can inhibit T98G cell growth to some degree; when MS-5 and MS-9 polysaccharide treatment concentrations are 5 mg/mL, the glioma cell viability levels are 45.81 and 47.31%, while the corresponding half effective concentrations are 2.153 mg/mL and 2.402 mg/mL, suggesting that their polysaccharides have weaker apoptosis-promoting effects.

## 4 Discussion

Liquid fermentation technology is necessary to produce edible fungi which can generate multiple active substances, including polysaccharides, flavone, and triterpenes, within a short time (Yan et al., 2023; Wei et al., 2022). Liquid fermentation technology is currently a research hotspot, both at home and abroad. The current research on *Phellinus igniarius* liquid fermentation culture mostly focuses on optimization, through adopting the orthogonal test or response surface methodology, with biomass and exopolysaccharides as target products. Tang et al. (2018) conducted an orthogonal test with *Phellinus igniarius*’ biomass and polysaccharide yields as indicators, in which case, the optimized media were 70 g saccharose, 10 g NH_4_CL, 1 g NaCL, 0.5 g KH_2_PO_4_, 0.5 g MgSO_4_, and 1,000 mL water. Zhou et al. (2017) conducted a study of different carbon sources and nitrogen sources, as well as their added proportions, arriving at the optimum media of 50 g soybean, 50 g niblets, 30 g glucose, and 1,000 mL water. This study also adopted a single-factor test and response surface methodology in order to identify the optimum fermentation medium formula for two *Inonotus hispidus* strains, as follows: the optimum fermentation conditions for MS-5 include 24.09 g/L glucose, 7.88 g/L yeast extract, 0.99 g/L dandelion powder, 1.5 g MgSO_4_, 2 g KH_2_PO_4_, 0.01 g vitamin B_1_, and 1 L deionized water; the optimum fermentation conditions for MS-9 include 24.64 g/L glucose, 7.77 g/L yeast extract, 0.98 g/L dandelion powder, 1.5 g MgSO_4_, 2 g KH_2_PO_4_, 0.01 g vitamin B_1_, and 1 L deionized water. It is predicted that, under such fermentation conditions, the mycelial biomass levels can reach 16.02 g/L and 14.91 g/L for MS-5 and MS-9, respectively. As revealed through a verification test, the optimized mycelial biomass levels for MS-5 and MS-9 are 1.6 and 1.54 times those before optimization; they significantly increase the liquid fermentation rate and are expected to be applied in factory production.

In order to gain insight into the underlying factors contributing to this enhanced fermentation rate, a detailed analysis was conducted to examine the effect of various carbohydrate concentrations and nitrogen sources on mycelial development. It is hypothesized that the impact of disparate carbohydrate and nitrogen sources on mycelial growth is contingent upon the capacity of the microorganisms in question to absorb and metabolize these substances.

The substantial promotion of mycelium development by glucose may be attributed to its status as a simple monosaccharide, which serves as a primary carbon source for microbial metabolism. The rapid absorption of glucose by microorganisms allows for its direct entry into the glycolysis pathway, thereby providing the mycelium with a rapid source of energy and a carbon skeleton for growth. Glucose is metabolized via the glycolysis pathway, which produces ATP and other essential metabolic intermediates. These metabolites can be used for the synthesis of new biomolecules, including proteins, lipids, and polysaccharides, thereby promoting the rapid growth of the mycelium. The utilization of glucose as a carbon source by fungi enables rapid growth and expansion in an environment where this process is advantageous due to the rapid and efficient metabolism of glucose.

Sucrose, lactose, and galactose have been demonstrated to have minimal growth-promoting effects, which is likely due to the fact that these sugars typically require hydrolysis or conversion by specific enzyme systems prior to utilization by microorganisms. Sucrose and lactose require hydrolysis by sucrase and lactase, respectively, prior to the production of usable monosaccharides. Galactose must undergo isomerization to glucose or other metabolic intermediates prior to entering the glycolysis pathway, a process that is relatively slow and complex. The longer metabolic pathway of these carbon sources necessitates the involvement of additional enzymes, resulting in greater energy consumption and a consequently weaker effect on mycelial growth promotion.

The addition of yeast extract and peptone has been demonstrated to exert a considerable promoting effect. Yeast extract and peptone are complex organic nitrogen sources that are rich in amino acids, vitamins, minerals, and other growth factors. These ingredients are readily absorbed and utilized by microorganisms, thereby providing a rich source of nutrients for the growth of mycelium. Furthermore, yeast extract may also provide trace elements and vitamins, thereby promoting additional growth. As fungi do not require complex metabolic conversions to utilize the rich nutrients in these nitrogen sources, these two nitrogen sources are the most effective at promoting growth. Beef extract has been demonstrated to exert a growth-promoting effect that is inferior to that of yeast extract and peptone. It is postulated that beef extract may contain slightly reduced concentrations of specific amino acids or growth factors in comparison to the latter two, which may result in a slightly diminished promoting effect.

Inorganic nitrogen sources, including ammonium tartrate, ammonium sulfate, and ammonium nitrate, have been demonstrated to exert minimal promoting effects. Although inorganic nitrogen sources provide nitrogen elements, they lack the amino acids and growth factors present in complex organic nitrogen sources. The conversion of inorganic nitrogen into amino acids requires additional metabolic steps and energy, as well as enzymatic reactions. This process is limited by the fact that it cannot be completed rapidly, which in turn limits the rapid growth of mycelium.

Among nitrogen sources, urea has been demonstrated to have the least favorable growth-promoting effect. Prior to being utilized by microorganisms, urea must undergo a conversion process into ammonia and carbon dioxide via the enzyme urease. The ammonia subsequently enters the nitrogen metabolism pathway. The conversion process is relatively slow, and at high concentrations, ammonia may be toxic to cells, thereby inhibiting growth.

In conclusion, single hexose sugars (such as glucose) can typically markedly enhance the growth of mycelium due to their straightforward metabolism. In contrast, complex sugars or pentose sugars necessitate more intricate metabolic pathways and, as a result, exert a comparatively weaker promoting effect. The utilization of complex organic nitrogen sources, such as yeast extract and peptone, provides a more nutritionally dense environment and can significantly enhance the growth of mycelium. Inorganic nitrogen sources and simple organic nitrogen sources (such as urea) have been observed to exert relatively weak promoting effects, which can be attributed to the necessity of additional metabolic steps.

As indicated in the antioxidant results for *Inonotus hispidus* exopolysaccharides, both MS-5 and MS-9 have high free radical scavenging abilities, though MS-9 has a higher free radical scavenging ability than MS-5 and exhibits better antioxidant activity. MS-5 and MS-9 have similar ABTS free radical scavenging abilities, and the EC_50_ values of MS-5 and MS-9 are 0.058 mg/mL and 0.056 mg/mL, respectively. MS-9 has a significantly higher DPPH free radical scavenging ability than MS-5, with an EC_50_ value of only 0.067 mg/mL. MS-5 and MS-9 are similar in total antioxidant capacity; when polysaccharide concentrations are 5 mg/mL, their FRAP values are 2.96 ± 0.15 and 3.53 ± 0.19, respectively. Therefore, *Inonotus hispidus* exhibits very high antioxidant activity and has potential value for development into antioxidant health products.

As revealed in an *in vitro* anticancer cell test on *Inonotus hispidus* exopolysaccharides, they produce the strongest apoptosis-promoting effects on human neurogliocytoma cells U251; with MS-9, glioma U251 cell viability is only 29.38%; in the case of MS-5, cell viability is 35.15%; in the case of MS-9, hepatoma carcinoma cell HepG-2 viability is 28.08%, while MS-5’s inhibition against it is moderate; MS-9’s inhibiting effect on colon cancer cell HCT-116 is significant, with a half effective concentration of only 0.963 mg/mL, but MS-5’s inhibiting effect is not significant; with MS-5, human prostatic cancer cell PC-3 viability is 37.32%, higher than that with MS-9. *Inonotus hispidus* has a weak inhibiting effect on the human breast cancer MCF-7 and glioma T98G cells, with cell viability levels reaching 43.49%–44.97 and 45.81%–47.31%, respectively. This study can serve as a reference for the development and utilization of exopolysaccharides for *Inonotus hispidus* fermentation broth.

## 5 Conclusion

Overall, this study adopted a single-factor test and the response surface methodology in order to optimize the liquid fermentation medium for two *Inonotus hispidus* strains, arriving at the optimum fermentation medium formula, in addition to revealing that *Inonotus hispidus* exopolysaccharides exhibit high *in vitro* antioxidant activity and very high antitumor activity, thus providing basic data for the development of natural antioxidant products, as well as the future research and development of anticancer drugs.

## Data Availability

The raw data supporting the conclusions of this article will be made available by the authors, without undue reservation.
